# Enhancing Health Literacy in Endourology: Using Artificial Intelligence to Improve Readability of Patient Education Materials

**DOI:** 10.7759/cureus.105155

**Published:** 2026-03-13

**Authors:** Loui Othman, Amar Kassim, Andrea Shehaj, Yahya Khan, Sri Manivasagam, Adam ElSayed, Claire Tse, Lakshay Khosla, Akin S Amasyali

**Affiliations:** 1 Department of Urology, Penn State College of Medicine, Hershey, USA; 2 Department of Emergency Medicine, Penn State College of Medicine, Hershey, USA; 3 Department of Neurosurgery, Penn State College of Medicine, Hershey, USA; 4 Department of Urology, Penn State Health Milton S. Hershey Medical Center, Hershey, USA; 5 Department of Orthopaedic Surgery, Penn State College of Medicine, Hershey, USA

**Keywords:** artificial intelligence, endourology, health literacy, patient education materials, readability

## Abstract

Purpose

Patient education materials (PEMs) enhance healthcare access and inclusivity, particularly for individuals without clinical backgrounds. However, many PEMs exceed recommended readability levels. This study evaluated whether artificial intelligence (AI)-assisted editing using ChatGPT-4o (OpenAI, San Francisco, CA, USA) could improve the readability of endourology PEMs related to prostate cancer, nephrolithiasis, bladder cancer, and kidney cysts.

Methods

Twenty-one publicly available PEMs from the American Urological Association (AUA) were analyzed. Each document was uploaded into ChatGPT-4o with instructions to rewrite the text to an eighth-grade reading level or lower while preserving content and word count. Readability of original and AI-modified PEMs was assessed using ReadabilityFormulas.com across six validated indices: Flesch-Kincaid Grade Level (FKGL), Simple Measure of Gobbledygook (SMOG), Gunning-Fog Index (GFI), Coleman-Liau Index (CLI), Automated Readability Index (ARI), and Flesch Reading Ease (FRE). Pre- and post-AI readability scores were compared using paired two-tailed t-tests.

Results

Across all indices and disease categories, AI modification significantly improved readability. Unmodified PEMs were written at approximately the ninth to 11^th^ grade level, whereas AI-modified versions were reduced to the fourth to sixth grade range (p < 0.001 for all comparisons). AI-modified PEMs also demonstrated reduced variability across readability scores, indicating improved standardization.

Conclusions

ChatGPT-4o significantly improved the readability of AUA endourology PEMs, aligning them with established health literacy recommendations. AI-assisted editing represents a scalable and standardized approach to improving patient comprehension and accessibility of urologic education materials.

## Introduction

Given the expanding array of medications, therapies, and surgeries available to patients, it is important to help patients understand their treatment choices. Educational materials can help translate complex medical terminology into simpler language for individuals without medical backgrounds. In the U.S., the average adult reads at an eighth-grade level, and over half have literacy skills below the sixth grade [1]. The American Medical Association (AMA) and National Institutes of Health (NIH) advise that patient education materials (PEMs) be written at a third- to seventh-grade level [2]. Keeping materials below the national literacy average enhances accessibility and ensures broader inclusivity.

Recent research has assessed the readability of patient education content. Kamath et al. (2020) reviewed 492 online resources covering 17 pain-related procedures, determining an average readability score of grade 12.1, ranging from 10.9 to 13, using software incorporating 10 quantitative readability metrics [2]. Likewise, Cheng et al. (2022) analyzed glaucoma-related patient materials with validated tools and found them written at a 10^th^- to 11^th^-grade level [3]. These findings are consistent with an earlier study by Eltorai et al. (2014), which reported a mean readability score of 10.9 ± 1.8 for patient education articles on the American Association for the Surgery of Trauma (AAST) website [4]. Within urology, widely available online PEMs for nephrolithiasis far exceed the recommended readability levels as outlined by the American Medical Association’s health literacy manual [5]. Collectively, these studies highlight the pressing need to simplify educational materials for greater patient accessibility.

Artificial intelligence (AI) technologies, such as ChatGPT (developed by OpenAI, San Francisco, CA, USA), have recently been explored for their potential to assist in medical writing, patient-facing communication, and health education. Prior research suggests that AI-generated text can simplify complex medical information while maintaining core content accuracy, making it attractive for the refinement of PEM. More broadly, recent analyses of AI in clinical medicine have highlighted its expanding role in supporting diagnostics, therapeutics, precision-guided treatments, and operational efficiency within healthcare systems [6]. Within this context, the application of AI to improve the readability and accessibility of PEMs represents a natural extension of these capabilities. The purpose of this study is to evaluate whether ChatGPT-4o can simplify urology PEMs on prostate cancer, nephrolithiasis, bladder cancer, and kidney cysts, thereby creating more accessible and inclusive resources for patients.

## Materials and methods

Study materials

A total of 21 PEMs were downloaded from the American Urological Association (AUA) website in June 2025 [7]. Materials were identified through the publicly accessible AUA patient education portal by reviewing all available resources related to endourologic conditions at the time of collection. The selection of relevant materials was conducted in consultation with practicing urologists with endourology experience to ensure clinical relevance. Inclusion criteria consisted of publicly available English-language PEMs addressing endourologic diseases or procedures. Duplicate materials, clinician-directed documents, and resources without substantial textual content were excluded. The final dataset comprised all eligible AUA patient education resources that met these criteria at the time of review.

The documents included multiple prostate cancer educational pamphlets, such as prostate cancer screening, salvage radiation therapy, prostatectomy, androgen deprivation therapy, genomic testing, and decision-making guidance materials. Additional materials addressed kidney stone prevention, diagnostic evaluation, stent education, calcium-related nephrolithiasis counseling, and general kidney stone educational content. Bladder cancer educational materials included prevention resources, pamphlets on muscle-invasive and non-muscle-invasive bladder cancer, and genetic testing information. Lastly, kidney cyst educational materials were also included to ensure comprehensive representation of endourology-related educational resources.

Each document was converted into a plain-text file. Following the approach of Kue et al., any institutional logos, disclaimers, or headers were removed while preserving medically relevant content, punctuation, and symbols [8].

AI-modified versions

To generate simplified versions of the educational materials, the original text of each pamphlet was input into ChatGPT-4o in July 2025. A standardized prompt was applied to each document to ensure consistent modification across materials.

Representative Prompt Example

A sample prompt is as follows: "Rewrite the file to ensure that the readability of the PEM is at an eighth-grade level or lower while maintaining the original medical meaning, word count, and clinical accuracy."

Representative Output Example

A representative excerpt of the original PEM and the corresponding AI-simplified version generated using this prompt is presented in Table 1.

**Table 1:** Comparison of original and AI-rewritten patient education text snippet AI: artificial intelligence

Original Text	AI-Simplified text
What is Salvage Radiation Therapy? Salvage radiation therapy (SRT) is a type of cancer treatment given to men who had their prostate removed, yet their prostate-specific antigen (PSA) levels start to rise. Biochemical recurrence is a term used to denote prostate cancer recurrence only detectable by a rising PSA level.	What is Salvage Radiation Therapy? Salvage radiation therapy (SRT) is a cancer treatment for men who have had their prostate removed and later have a rise in their prostate-specific antigen (PSA) level. A rising PSA after surgery may mean the cancer has returned. When prostate cancer is found only by a rising PSA level, this is called biochemical recurrence.

Readability analysis

The cleaned text from both original and AI-modified pamphlets was then scored using six different readability assessments. All readability tools used in this study are publicly available and open-access. No licensing or permission requirements were required for their application in this study.

Readability scores were calculated using the free online tool available at ReadabilityFormulas.com, which allowed for consistent calculation across all indices. This website has been validated in multiple published readability analyses and is frequently used in peer-reviewed studies evaluating PEMs across medical specialties [9-12].

The six readability assessments used for this analysis included the following grades.

Flesch-Kincaid Grade Level (FKGL)

FGKL provides an equivalent U.S. school grade level derived from the same inputs as Flesch-Kincaid Reading Ease (FRE) (sentence length and syllable count) [13].

$\mathrm{FKGL} = 0.39\left(\frac{\mathrm{words}}{\mathrm{sentences}}\right) + 11.8\left(\frac{\mathrm{syllables}}{\mathrm{words}}\right) - 15.59$

Simple Measure of Gobbledygook (SMOG)

SMOG estimates the reading grade level by counting polysyllabic words. SMOG was particularly cited to be useful to health literacy guidelines issued by the National Cancer Institute [14]. 

$\mathrm{SMOG} = 1.043 \sqrt{\frac{\text{polysyllabic words} \times 30}{\mathrm{sentences}}} + 3.1291$

Gunning-Fog Index (GFI)

The GFI Measures readability based on average sentence length and percentage of complex words (three or more syllables) [15].

$\mathrm{GFI} = 0.4 \left[ \left(\frac{\mathrm{words}}{\mathrm{sentences}}\right) + 100\left(\frac{\text{complex words}}{\mathrm{words}}\right) \right]$

Coleman-Liau Index (CLI)

The CLI is based on characters per word rather than syllables [16].

$\mathrm{CLI} = 0.0588\left(\frac{\mathrm{letters}}{100\,\mathrm{words}}\right) - 0.296\left(\frac{\mathrm{sentences}}{100\,\mathrm{words}}\right) - 15.8$

Automated Readability Index (ARI)

Like CLI, the ARI also relies on characters rather than syllables [17].

$\mathrm{ARI} = 4.71\left(\frac{\mathrm{characters}}{\mathrm{words}}\right) + 0.5\left(\frac{\mathrm{words}}{\mathrm{sentences}}\right) - 21.43$

The FRE Score

The FRE score assesses readability on a 0-100 scale based on average sentence length and average syllables per word. Higher FRE scores indicate easier text (e.g., a score ≥80 suggests ~sixth-grade level, "easy" readability), whereas lower scores indicate more difficult text (e.g., a score ~30 corresponds to college-level complexity) [18]. 

$\mathrm{FRE} = 206.835 - 1.015\left(\frac{\mathrm{words}}{\mathrm{sentences}}\right) - 84.6\left(\frac{\mathrm{syllables}}{\mathrm{words}}\right)$

For each educational document, readability scores from six validated indices were systematically recorded and compared against established benchmarks recommended by the AMA and the NIH. These organizations advise that PEMs should be written at or below a sixth-grade (AMA) or eighth-grade (NIH) reading level to optimize patient comprehension [9,19]. Each readability score was interpreted according to U.S. grade level and categorized into qualitative reading levels using standardized descriptors.

Previous studies primarily utilized FKG and FKE indices and focused on a sixth-grade cutoff; this approach was expanded to include four additional measures: the GFI, CLI, SMOG, and ARI. Employing multiple established readability formulas enhances reliability, as each formula evaluates readability using distinct parameters such as sentence length, syllable count, polysyllabic word frequency, complex word percentage, or character-to-word ratios. This comprehensive methodology aligns with best practices in readability assessment as described in recent systematic reviews and specialty society analyses [6,19-23].

Statistical analysis

For each urology PEM, descriptive statistics were calculated, including the mean and standard deviation of readability scores across all six indices. To evaluate the effect of AI modification, pre-AI and post-AI readability scores were compared using paired two-tailed t-tests, as the same set of materials was assessed before and after AI modification. A p-value less than 0.05 was considered statistically significant.

## Results

**Figure 1:**
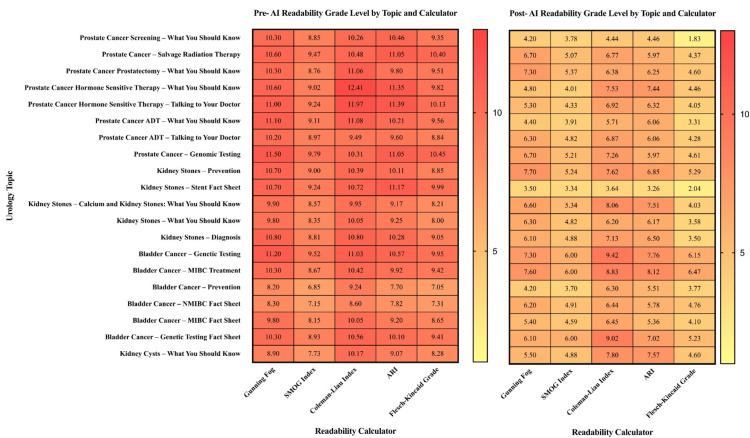
Heatmap showing pre-AI and post-AI readability grade levels across urology topics and six validated readability calculators. AI: artificial intelligence; SMOG: Simple Measure of Gobbledygook; ARI: Automated Readability Index; ADT: androgen deprivation therapy; MBIC: muscle-invasive bladder cancer; NMIBC: non-muscle-invasive bladder cancer

Before GPT-4o modification, the AUA PEMs demonstrated consistently high reading complexity across all six readability metrics. Average GFI scores were in the 9 to 11 range, with SMOG scores between about 8 and 9 and FKG around 8 to 10, placing all materials at or above a ninth- to 10^th^-grade reading level. The CLI and ARI showed nearly identical patterns, averaging around 10 across topics. FRE scores were low, hovering near 55 to 60, which corresponds to “fairly difficult” readability. These findings placed the original PEMs well above the AMA- and NIH-recommended sixth-grade threshold for PEMs (Figure 1).

The heatmap of pre- and post-AI readability (Figure 1) highlights these differences across all 21 individual pamphlets. Pre-AI, nearly every topic clustered around the ninth to 11^th^ grade level, regardless of the index used, with darker red shading indicating high textual complexity. After GPT-4o modification, the heatmap shifted to lighter yellow and orange tones across every index, reflecting grade levels consistently reduced to between fourth and sixth grade. Notably, prostate cancer materials, which were among the most difficult to read pre-AI, demonstrated the greatest improvements. Kidney stone and kidney cyst pamphlets started closer to the recommended threshold, but still showed clear downward shifts in grade level. This visual pattern underscores the uniform effect of AI modification across different disease categories and readability formulas.

Quantitatively, GPT-4o modification improved readability substantially and uniformly. GFI scores decreased by about half, dropping from 9 to 11 to approximately 5 to 6 across all topics (for example, 10.70 ± 0.46 to 5.71 ± 1.18 in prostate cancer, p < 0.001). SMOG scores fell from around 8 to 9 to 4 to 5 (for example, 9.15 ± 0.34 to 4.56 ± 0.63, p < 0.001). The CLI declined from averages near 10 to about 6 (for example, 10.88 ± 0.96 to 6.48 ± 0.99, p < 0.001), and ARI dropped from similar levels to around 5 to 6 (for example, 10.61 ± 0.70 to 6.07 ± 0.81, p < 0.001).

FRE scores rose sharply, reflecting this drop in complexity. Whereas pre-AI values were mostly in the mid-50s, post-AI values climbed to the mid-70s and low-80s, representing a shift from “fairly difficult” to “standard” or “fairly easy” readability (for example, 55.75 ± 3.41 to 76.62 ± 6.61, p < 0.001). FKG scores mirrored these results, falling from about ninth-grade to near fifth-grade levels (for example, 9.72 ± 0.58 to 4.93 ± 0.52, p < 0.001).

**Figure 2:**
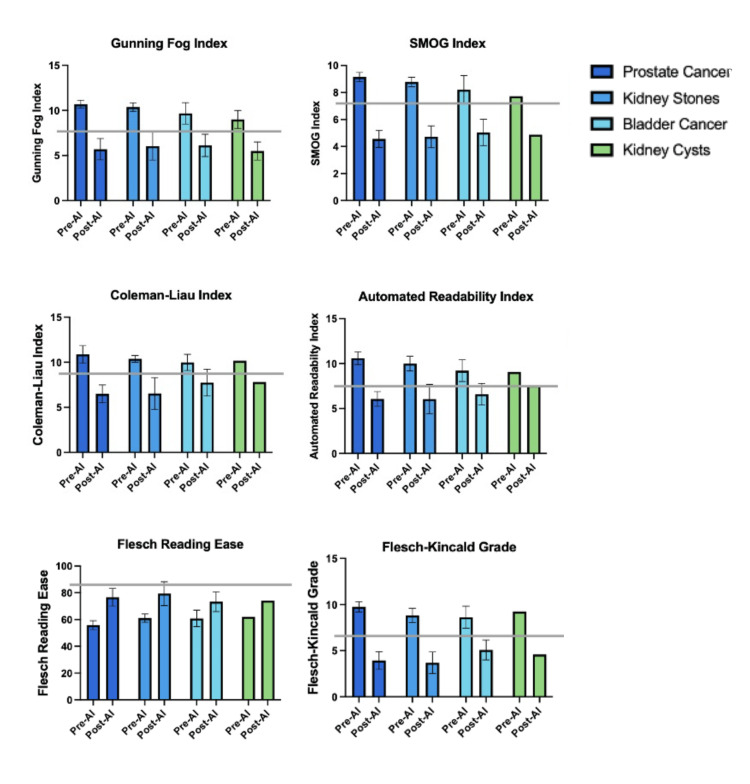
Grouped bar graphs displaying pre-AI versus post-AI readability indices across four urology topic categories. AI: artificial intelligence; SMOG: Simple Measure of Gobbledygook

The bar graph comparisons (Figure 2) reinforce these improvements at the category level. Across prostate cancer, kidney stones, bladder cancer, and kidney cyst PEMs, every index demonstrated significant reductions in complexity after GPT-4o modification. Prostate cancer PEMs, initially the most complex, showed the largest absolute decreases, while kidney stone and cyst materials still benefited, but with smaller relative changes. Prostate cancer, kidney stones, bladder cancer, and kidney cyst PEMs: every index demonstrated significant reductions in complexity after GPT-4o modification. Prostate cancer PEMs, initially the most complex, showed the largest absolute decreases, while kidney stone and cyst materials still benefited but with smaller r.

Importantly, variability narrowed considerably after GPT-4o modification. The standard deviation of FKG scores decreased from about 1.0 pre-AI to 0.3 post-AI, and similar patterns were seen for the other metrics, indicating that GPT-4o produced PEMs with far more uniform readability than the original AUA materials. Overall, GPT-4o reduced the reading level of all 21 PEMs by approximately four grade levels and brought them into alignment with national recommendations for patient-facing content.

## Discussion

The findings of this study show that GPT-4o can substantially enhance the readability of endourology PEMs, reducing their reading level from well above the recommended threshold to a level appropriate for the majority of the U.S. adult population. As shown in Figure 1, all pre-AI readability scores clustered around the ninth to 11^th^ grade range across five different metrics, while post-AI scores dropped to approximately the fourth to sixth grade level for every topic. This effect was consistent across all readability metrics, with every post-AI value falling below the sixth-grade benchmark (Figures 1-2). This is particularly significant given that approximately 54% of U.S. adults possess literacy skills below a sixth-grade level [24]. Limited health literacy is a strong predictor of adverse health outcomes and contributes to persistent health disparities [25]. By raising the readability of PEMs, AI-driven editing could help bridge this gap and make critical health information more accessible to patients with lower literacy skills.

The pre-AI readability levels observed in this study are consistent with a large body of literature showing that PEMs across medical specialties frequently exceed recommended reading levels. As shown in Figure 2, the mean pre-AI values for all endourology subtopics (prostate cancer, kidney stones, bladder cancer, and kidney cysts) were clustered well above the horizontal benchmark line at sixth grade, similar to what has been documented in orthopedics [10], gynecologic oncology [26], rheumatology [19], vascular surgery [9,23,27], and chronic venous disease [28]. Even patient-facing content published by high-impact journals is typically written well above the sixth-grade level, with only 2.1% of materials meeting this threshold over 20 years [29]. Furthermore, a systematic review spanning more than 29,000 PEMs across 438 studies found that readability levels have not improved in the past three decades despite growing awareness of health literacy challenges [30]. The present study adds to this literature by demonstrating that the challenge is similarly pervasive in endourology and by showing that it can be effectively addressed using AI.

Improving readability is not merely a technical endeavor. Health literacy directly affects patients’ ability to understand their diagnoses, adhere to treatment regimens, and make informed decisions about their care [31,32]. Endourology patients often must navigate complex management pathways involving procedural options, post-operative care, and long-term surveillance. Inadequate comprehension of these topics can compromise adherence and outcomes, particularly among older adults or those with lower baseline health literacy [24,33]. By lowering reading levels from approximately 10^th^ grade to fifth to sixth grade across all topics (Figure 1), GPT-4o may help reduce this cognitive burden, promote shared decision-making, and support health equity by ensuring that patients from all educational backgrounds can access and use essential information [19,25].

A notable strength of this approach is its scalability. GPT-4o produced simplified versions of all 21 PEMs with remarkable consistency, as evidenced by the narrowing standard deviations shown across post-AI scores in Figure 2. This suggests that AI can provide a standardized baseline of readability, minimizing the variability introduced by individual authorship or editing styles [34]. In practice, scalable implementation would involve AI generating readability-optimized drafts that are subsequently reviewed and approved by clinicians before distribution to patients. This approach enables rapid readability improvement while maintaining clinical oversight and ensuring that essential medical information is preserved. Prior work has shown that manual editing to achieve target reading levels can be labor-intensive and inconsistently applied [35]. By contrast, AI can achieve this quickly while maintaining topic integrity. Although some studies have raised concerns that simplification may risk omitting critical information [36], our findings suggest that GPT-4o maintained essential content while improving clarity, as evidenced by consistent improvements across all PEMs (Figures 1-2) without erasing key concepts.

Despite these limitations, this study provides compelling evidence that AI can be a powerful tool for improving the accessibility of patient-facing materials in endourology. The consistent and substantial reduction in reading level achieved by GPT-4o (Figures 1-2) demonstrates its potential to standardize the readability of PEMs, thereby improving health literacy and supporting informed decision-making. As professional societies increasingly recognize their responsibility to promote equitable care, integrating AI-based readability editing into PEM development represents a promising and actionable strategy.

Limitations

Several limitations should be acknowledged. First, this study analyzed a relatively small sample of PEMs and relied exclusively on AUA PEMs for four endourology conditions. As a result, the findings may reflect sampling bias related to a single professional organization's educational content and may not represent the full range of urologic or broader medical educational materials available to patients. Second, readability indices measure textual complexity but do not directly assess patient comprehension, decision-making, or health outcomes. Third, figures, diagrams, and multimedia content, which are often important for patient understanding, were not included in the analysis because the source materials were text-only. Fourth, the study was restricted to English-language materials, limiting its generalizability to patients who require non-English resources. Finally, while GPT-4o was effective, AI technology continues to evolve rapidly, and future models may yield different outputs. Future work should incorporate comprehension testing in diverse patient populations, clinician validation of AI-modified content, and application across multilingual PEMs.

## Conclusions

This study demonstrates that GPT-4o can substantially reduce the reading grade level of PEMs across multiple validated readability indices. These findings suggest that AI-assisted editing may offer a scalable approach to improving the accessibility of PEMs. However, this analysis evaluated readability rather than patient comprehension or clinical usability. Future studies should evaluate whether AI-modified materials improve patient comprehension and decision-making, incorporate clinician review to ensure content accuracy, and examine implementation across broader and multilingual patient education resources. 

## References

[1] Miller B, McCardle P, Hernandez R (2010). Advances and remaining challenges in adult literacy research. J Learn Disabil.

[2] Kamath D, Mcintyre S, Peskin E (2020). Readability of online patient education materials for interventional pain procedures. Cureus.

[3] Cheng BT, Kim AB, Tanna AP (2022). Readability of online patient education materials for glaucoma. J Glaucoma.

[4] Eltorai AE, Ghanian S, Adams CA Jr, Born CT, Daniels AH (2014). Readability of patient education materials on the American Association for Surgery of Trauma website. Arch Trauma Res.

[5] Bergersen AM, Khan I, Wong AC, Chipollini JJ, Weiss BD, Tzou DT (2021). Online kidney stone educational materials do not meet recommended readability standards. Urol Pract.

[6] Sallam M, Snygg J, Allam D, Kassem R, Damani M (2025). Artificial intelligence in clinical medicine: a SWOT analysis of AI progress in diagnostics, therapeutics, and safety. J Innov Med Res.

[7] (2025). American Urological Association: educational resources. https://www.urologyhealth.org/educational-resources?product_format=466%7C&language=1122%7C.

[8] Kue J, Klemanski DL, Browning KK (2021). Evaluating readability scores of treatment summaries and cancer survivorship care plans. JCO Oncol Pract.

[9] Bailey MA, Coughlin PA, Sohrabi S, Griffin KJ, Rashid ST, Troxler MA, Scott DJ (2012). Quality and readability of online patient information for abdominal aortic aneurysms. J Vasc Surg.

[10] Restrepo M, Stern BZ, Burnett GW, Park C, Poeran J (2025). The readability of online English and Spanish patient education materials on anaesthesia for orthopaedic surgery. BJA Open.

[11] Eltorai AE, Sharma P, Wang J, Daniels AH (2015). Most American Academy of Orthopaedic Surgeons' online patient education material exceeds average patient reading level. Clin Orthop Relat Res.

[12] Badarudeen S, Sabharwal S (2010). Assessing readability of patient education materials: current role in orthopaedics. Clin Orthop Relat Res.

[13] Flesch R (1948). A new readability yardstick. J Appl Psychol.

[14] McLaughlin HG (1969). SMOG grading-a new readability formula. J Read.

[15] Gunning R (1952). The Technique of Clear Writing. No Title.

[16] Coleman M, Liau TL (1975). A computer readability formula designed for machine scoring. J Appl Psychol.

[17] Senter RJ, Smith EA (1966). Automated Readability Index. https://apps.dtic.mil/sti/html/tr/AD0667273/.

[18] Kincaid JP, Fishburne Jr RP, Rogers RL, Chissom BS (1975). Derivation of New Readability Formulas (Automated Readability Index, Fog Count and Flesch Reading Ease Formula) for Navy Enlisted Personnel. https://apps.dtic.mil/sti/html/tr/ADA006655/.

[19] Rustomji Y, Nweke UC, Hassan S, Ahmad U, Jolly M (2025). Not so patient friendly: patient education materials in rheumatology and internal medicine fall short of nationally recommended readability benchmarks in the United States. Arthritis Care Res (Hoboken).

[20] Hanci V, Otlu B, Biyikoğlu AS (2024). Assessment of the readability of the online patient education materials of intensive and critical care societies. Crit Care Med.

[21] Fahimuddin FZ, Sidhu S, Agrawal A (2019). Reading level of online patient education materials from major obstetrics and gynecology societies. Obstet Gynecol.

[22] van Ballegooie C, Hoang P (2021). Assessment of the readability of online patient education material from major geriatric associations. J Am Geriatr Soc.

[23] Scott BB, Johnson AR, Doval AF, Tran BN, Lee BT (2020). Readability and understandability analysis of online materials related to abdominal aortic aneurysm repair. Vasc Endovascular Surg.

[24] Stormacq C, Van den Broucke S, Wosinski J (2019). Does health literacy mediate the relationship between socioeconomic status and health disparities? Integrative review. Health Promot Int.

[25] Berkman ND, Sheridan SL, Donahue KE, Halpern DJ, Crotty K (2011). Low health literacy and health outcomes: an updated systematic review. Ann Intern Med.

[26] Samuel D, Vilardo N, Isani SS, Kuo DY, Gressel GM (2019). Readability assessment of online gynecologic oncology patient education materials from major governmental, non-profit and pharmaceutical organizations. Gynecol Oncol.

[27] Avra TD, Le M, Hernandez S, Thure K, Ulloa JG (2022). Readability assessment of online peripheral artery disease education materials. J Vasc Surg.

[28] Liao J, Wu Z, Zhao J (2023). A readability analysis of patient education materials about chronic venous disease provided by professional vascular societies. Phlebology.

[29] Rooney MK, Santiago G, Perni S (2021). Readability of patient education materials from high-impact medical journals: a 20-year analysis. J Patient Exp.

[30] Okuhara T, Furukawa E, Okada H, Yokota R, Kiuchi T (2025). Readability of written information for patients across 30 years: a systematic review of systematic reviews. Patient Educ Couns.

[31] Strijbos RM, Hinnen JW, van den Haak RF, Verhoeven BA, Koning OH (2018). Inadequate health literacy in patients with arterial vascular disease. Eur J Vasc Endovasc Surg.

[32] Weiss BD (2003). Health Literacy: A Manual for Clinicians: Part of an Educational Program About Health Literacy. http://lib.ncfh.org/pdfs/6617.pdf.

[33] Savji N, Rockman CB, Skolnick AH, Guo Y, Adelman MA, Riles T, Berger JS (2013). Association between advanced age and vascular disease in different arterial territories: a population database of over 3.6 million subjects. J Am Coll Cardiol.

[34] Josfeld L, Huebner J (2023). Development and analysis of quality assessment tools for different types of patient information - websites, decision aids, question prompt lists, and videos. BMC Med Inform Decis Mak.

[35] Mac O, Ayre J, Bell K, McCaffery K, Muscat DM (2022). Comparison of readability scores for written health information across formulas using automated vs manual measures. JAMA Netw Open.

[36] Ivchenko O, Grabar N (2022). Impact of the text simplification on understanding. Stud Health Technol Inform.

